# Comparison of DNA Extracted from Pediatric Saliva, Gingival Crevicular Fluid and Site-Specific Biofilm Samples

**DOI:** 10.3390/mps3030048

**Published:** 2020-07-09

**Authors:** Jason Emett, Roxanne David, Jaydene McDaniel, Steven McDaniel, Karl Kingsley

**Affiliations:** 1 Department of Clinical Sciences, University of Nevada, Las Vegas—School of Dental Medicine, 1700 W. Charleston Blvd, Las Vegas, NV 89106, USA; emett@unlv.nevada.edu (J.E.); davidr9@unlv.nevada.edu (R.D.); 2 DMD—Department of Advanced Education in Pediatric Dentistry, University of Nevada, Las Vegas—School of Dental Medicine, 1700 W. Charleston Blvd, Las Vegas, NV 89106, USA; mcdanj1@unlv.nevada.edu (J.M.); mcdans1@unlv.nevada.edu (S.M.); 3 Department of Biomedical Sciences and Director of Student Research, University of Nevada, Las Vegas—School of Dental Medicine, 1001 Shadow Lane, Las Vegas, NV 89106, USA

**Keywords:** saliva screening, gingival crevicular fluid, oral biofilm, paper point sampling, DNA isolation

## Abstract

(1) Introduction: Due to the non-invasive nature of saliva, many methods have been used to isolate and collect DNA from saliva samples for microbial screening. Many oral microbes also inhabit the oral biofilm, which may represent significantly different microbial constituents that may contribute to oral health and disease, including caries and periodontal disorders. Moreover, the biofilm may vary within the same patient at different sites. Few studies have evaluated the comparison between DNA isolated from saliva and DNA from site-specific biofilm, with virtually no studies addressing this analysis among pediatric patients. (2) Methods: An existing repository of paper point derived biofilm, gingival crevicular fluid (GCF), and unstimulated saliva samples previously collected from pediatric patients (*n* = 47) was identified. DNA was isolated from biofilm sites (tongue, upper buccal molar, mandibular lingual incisor), and GCF and saliva were used for quantitative DNA comparison using a phenol:chloroform extraction. A quantitative and qualitative analysis was performed using the NanoDrop 2000 spectrophotometer using absorbance readings at A230 nm, A260 nm and A280 nm. (3) Results: These data demonstrated the successful isolation of DNA from all of the patient samples, with the highest concentrations observed among unstimulated saliva (4264.1 ng/μL) and the lowest derived from GCF (1771.5 ng/μL). No differences were observed between males and females or minorities and non-minority patients. In addition, comparison of the overall concentrations of DNA obtained from adult samples was slightly higher than, but not significantly different from, the concentrations obtained from pediatric samples (*p* = 0.2827). A real-time quantitative qPCR screening revealed that all of the samples evaluated harbored bacterial and human DNA of sufficient quantity and quality for a molecular screening greater than the limit of detection (ΔRn = 0.01). (4) Conclusions: Many methods are currently available to provide the sampling and screening of saliva and specific sites within the oral cavity, but the validation and comparison of simple and low-cost methods, that include paper point sampling and unstimulated saliva collection, may suggest these methods and protocols provide sufficient DNA quality and quantity for molecular screening and other comparison applications. In addition, although heterogeneity will be a constant and consistent feature between patient samples, standardized methods that provide similar and consistent DNA from various oral sites may provide needed consistency for screening and molecular analysis.

## 1. Introduction

Due to the non-invasive nature of saliva collection, many methods have been used to isolate and collect DNA from saliva samples for microbial screening [1,2,3]. Screening methods for these samples have included highly specific and technical procedures involving immunofluorescence microscopy, proteomic analysis and next-generation sequencing [3,4,5,6]. Among the most sensitive, rapid, reliable and widely available methods for oral microbial screening is the polymerase chain reaction (PCR) [7,8]. Although a few studies have made detailed analyses from DNA isolated from saliva and other oral fluids, such as gingival crevicular fluid (GCF), as well as oral biofilm comparisons and analyses [9,10], to date there have been virtually no comparisons with oral site-specific biofilms to determine the comparative quality and quantity for a subsequent microbial PCR screening [11].

Many oral microbes also inhabit the oral biofilm, which may represent significantly different microbial constituents that may contribute to oral health and disease [12,13]. The production and growth of oral biofilms are implicated in oral diseases, including caries and periodontal disorders [14,15]. Therefore, the sampling and analysis of oral biofilms may also represent a significant source of readily available, non-invasive data regarding oral health and disease [16,17].

Although a previous study from this group evaluated the comparison between DNA isolated from saliva or GCF with DNA from site-specific biofilm in adults [11], virtually no studies addressing this analysis among pediatric patients have been completed. Based upon this lack of analytic data, the primary goal of this study was to make a quantitative and qualitative comparison to determine the suitability of the DNA from these oral samples for microbial PCR screening.

## 2. Methods

### 2.1. Study Approval

This was a retrospective analysis of previously collected saliva and oral samples. The protocol for this study was reviewed and approved exempt by the University of Nevada Las Vegas Office for the Protection of Research Subjects (OPRS) on 6 February 2015 under Protocol#1502-506M. The original protocol OPRS#1305-4466M “The Prevalence of Oral Microbes in Saliva from the UNLV School of Dental Medicine Pediatric and Adult Clinical Population” was approved on 31 May 2013. These protocols (and OPRS approval) comply with the Health Information Portability and Accountability Act (HIPAA) standards for the privacy of individually identifiable health information (Privacy Rule), issued by the United States Department of Health and Human Services (HHS).

### 2.2. Original Sample Collection

From the original protocol, patients (or parents/guardians if the patient was under 18 years of age) were asked for their voluntary participation. Any patient or parent/guardian that declined to participate was excluded. Patients were asked to provide informed consent. If patients were under 18 years of age, patients were asked to provide pediatric assent with the parent/guardian providing informed consent. No patients were given money or services for their participation.

Each patient was then given a sterile saliva collection tube and were requested to produce up to five mL of unstimulated saliva. Gingival crevicular fluid was acquired from the buccal gingival crevice of the maxillary central incisor using sterile paper points (Size 40 Quality Endodontic Distributors, QED) for a standardized three minutes. Biofilm samples were acquired from the dorsum of the tongue, supragingival biofilm on the buccal surface of the first upper molar (maxillary), and lingual surface of the central incisor (mandibular), as previously described [11]. Each sample was stored on ice and transferred to a biomedical biosafety level 2 (BSL-2) laboratory for long-term storage and processing. Samples from each patient were given a randomly generated, non-duplicated number to avoid any specific patient information or other identifying information from being associated with each sample. Only basic demographic information, such as age, sex and race/ethnicity, was noted.

### 2.3. DNA Isolation

Samples were thawed and DNA was isolated using the phenol:choloform extraction method with TRIzol reagent (Invitrogen) specifically designed to isolate high-quality nucleic acids (RNA, DNA) from tissues or fluids, as previously described [18,19]. In brief, 100 μL of thawed saliva was removed for analysis. For each paper point sample, 100 μL of sterile 1X phosphate buffered saline (PBS) was added to each paper point containing sample collection tubes and was vortexed to release any microbial or human cells. This solution was added to 300 μL of TRIzol reagent prior to incubation. After five minutes, 200 μL of chloroform was added and incubated for an additional five minutes. Each sample was subsequently centrifuged at 12,000× *g,* or the relative centrifugal force (RCF), for fifteen minutes at 4 °C.

The nucleic acid-containing phase was transferred to a new microcentrifuge tube with the addition of 100% isopropanol, which was gently mixed to precipitate the nucleic acids from the solution. These samples were then centrifuged for an additional five minutes to pellet the DNA. The supernatant was aspirated and the pellet washed with 100% ethanol and was centrifuged for an additional five minutes. The ethanol was aspirated and the DNA pellet was resuspended in 100 μL of DNA rehydration solution for analysis. Negative controls for liquid samples, including saliva and GCF (distilled water), as well as for paper points (sterile/blank paper points), were used where appropriate.

### 2.4. DNA Analysis

The concentration and purity of the DNA was determined using the NanoDrop spectrophotometer (ThermoFisher) with absorbance readings at 260 and 280 nm. An estimation of the DNA concentration can be obtained using the absorbance at A260 nm and the correction for turbidity at A320 nm, adjusting for the dilution factor. An estimation of the purity can be determined from the ratio of A260 nm and A280 nm with the corresponding dilution factor. High quality DNA will correspond with a ratio of A260:A280, ranging between 1.7–2.0. Absorbance at A230 was also measured to provide the A260:A230 ratio—commonly accepted as an accurate measure of the residual chemical contamination from nucleic acid extraction procedures. Expected values typically range from approximately 2.0 to 2.2 for “pure” nucleic acid samples acceptable for qPCR analysis and screening.

### 2.5. qPCR Screening

Screening for the presence of microbial and human DNA was accomplished using a quantitative polymerase chain reaction (qPCR) using primers for bacterial 16S rRNA and human glyceraldehyde 3-phosphate dehydrogenase (GAPDH), with specifications that included an initial incubation at 50 °C for two minutes, followed by denaturation at 95 °C for 10 min, and 25 cycles: denaturation at 95 °C for 15 s and annealing at 51 °C for one minute. The reaction included: 15 μL TaqMan universal PCR master mix (Applied Biosystems), 0.6 μL of the following primers at a concentration of 10 uM from Eurofins MWG Operon (Huntsville, AL), and 0.75 μL of probe resulting in a final probe concentration of 0.2 uM, with 2 μL of the DNA samples. Sterile, nuclease-free distilled water from Promega (Madison, WI, USA) was used to adjust the final reaction volume to 30 μL. Each screening was performed in duplicate.

The primers synthesized from Eurofins MWG Operon (Huntsville, AL, USA) were:

(Nucleotide = nt; Tm = melting temperature)
Bacterial 16S rRNA
Forward 16S rRNA universal primer, 5′-ACGCGTCGACAGAGTTTGATCCTGGCT-3′
27 nt, 56% GC, Tm 76 °C
Reverse 16S rRNA universal primer, 5′-GGGACTACCAGGGTATCTAAT-3′
21 nt, 48% GC, Tm 62 °C
16S probe: (6-FAM)-5′-CGTATTACCGCGGCTGCTGGCAC-3′-(TAMRA)
23 nt, 65% GC, Tm 76 °C

Human glyceraldehyde 3-phosphate dehydrogenase (GAPDH)

Forward primer-GAPDH, 5′-ATCTTCCAGGAGCGAGATCC-3′
20 nt, 55% GC, Tm 66 °C
Reverse primer-GAPDH, 5′- ACCACTGACACGTTGGCAGT-3′
20 nt, 55% GC, Tm 70 °C
GAPDH probe: (6-FAM)-5′-CCTCTACTGGCGCTGCCAAGGCT-3′-(TAMRA)
23 nt, 65% GC, Tm 77 °C

### 2.6. qPCR Analysis

Results from the real-time qPCR screening were analyzed using the delta (Δ) normalized reported (Rn) value or ΔRn. To obtain this value, the fluorescent signal from each qPCR reaction is normalized to the signal from the passive (unincorporated) reference dye by dividing the qPCR signal by the passive reference dye signal. The delta Rn value is then obtained by finding the difference between the normalized Rn value minus the baseline signal generated by the specific qPCR instrument. This analysis can be used to reliably calculate the magnitude of the specific signal produced by any given set of qPCR conditions above the threshold limit of detection (ΔRn = 0.01).

### 2.7. Statistical Analysis

DNA measurements involve continuous parametric data, therefore descriptive statistics regarding these data were given where appropriate (mean and the standard error of the mean (SEM)). Graphic analysis of data was visualized using box-and-whisker plots, which included using the Tukey method to plot the interquartile range (IQR) between the 25th and 75th percentiles, as previously described [11]. Any differences between the concentrations or purity between the samples or oral sites were measured using Student’s t-tests, which are appropriate for the analysis of parametric data. Due to the confounding influence of multiple two-way t-tests, the analysis of variance (ANOVA) was used to confirm all statistical analyses.

A demographic analysis of the study sample was performed using simple descriptive statistics (raw number and percentage of the overall sample) for comparison with overall clinic averages using the categorical variables (sex, race/ethnicity). Differences between categorical variables can be most appropriately analyzed using Chi Square (χ^2^) analysis, reporting the degrees of freedom (d.f.) and resulting *p*-values.

## 3. Results

A quantitative and qualitative analysis of DNA was successfully completed from all the cryopreserved patient samples, *n* = 47 (Table 1). The oral sites with the overall highest DNA concentrations were derived from unstimulated saliva (average 4264.1 ng/μL), which were significantly different from all other sites (*p* = 0.0348). All three of the sites sampling biofilm (the dorsum tongue, upper buccal and mandibular lingual) had averages (2382.4 ng/μL, 20148.4 ng/μL and 2428.6 ng/μL, respectively) that were not significantly different from one another (*p* = 0.5376). The oral site with the overall lowest DNA concentrations were derived from the gingival crevicular fluid (GCF; average 1771.5 ng/μL), which was not significantly different from those derived from the biofilm (*p* = 0.4805). In addition, DNA purity was measured using A260:A280 ratios, which was fairly consistent among all the sampling sites with averages ranging between 1.71 and 1.82. Because some individual samples had A260:A280 ratios as low as 1.55, absorbance at A230 was also measured to provide the A260:A230 ratio–commonly accepted as an accurate measure of the residual chemical contamination from nucleic acid extraction procedures. These results demonstrated a fairly low residual contamination, with values ranging from 1.92 to 2.01 that closely approximates the expected values between 2.0 and 2.2 for “pure” nucleic acid samples acceptable for qPCR analysis and screening.

**Figure 1:**
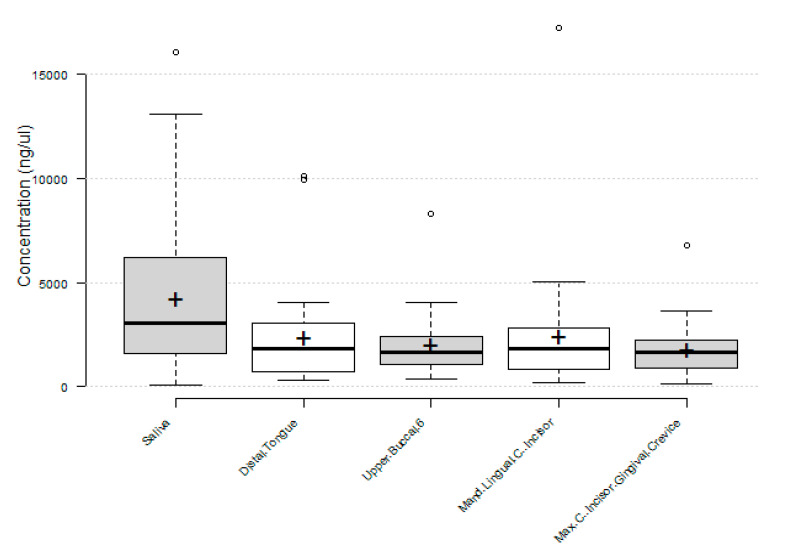
Box-and-whisker plot analysis of DNA from oral sampling sites. The Tukey method used to plot the interquartile range (IQR) between the 25th and 75th percentile reveals similarities between the DNA concentrations derived from biofilm and GCF, which were significantly lower than those derived from unstimulated saliva (*p* = 0.0348).

To visualize these data, box-and-whisker plots were created to evaluate the minimum, maximum, first and third quartile ranges (Figure 1). These data clearly demonstrate the similarity between the median and means of the non-salivary samples revealed by the data from Table 1. In addition, this demonstrated further similarity between the first quartile ranges of the tongue, upper buccal, mandibular lingual and GCF (713.4–1057.0 ng/μL) and third quartiles ranges (2214.6–3055.1 ng/μL), which were markedly different from those of unstimulated saliva (1588.2–6198.8 ng/μL).

**Figure 2:**
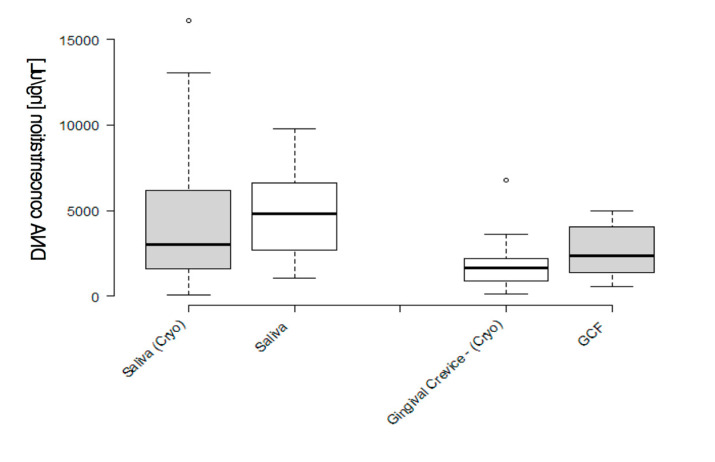
Comparison of DNA concentrations isolated from the frozen and original (fresh) saliva samples. A box-and-whisker plot analysis of data demonstrates a broader range of DNA concentrations among frozen samples when compared with fresh samples, with roughly similar averages (4264.1 ng/μL and 4809.3 ng/μL, *p* = 0.5389), and gingival crevicular fluid (1762 ng/μL and 2639.5 ng/μL, *p* = 0.2253).

To evaluate whether the results obtained from the cryopreserved samples were similar to the results obtained from the fresh samples taken during the same study [11], the average concentrations from the original samples (fresh) were plotted against those from the current study (frozen) to determine if the equivalent saliva sample volumes yielded similar amounts of DNA (Figure 2). These data demonstrated that, although the overall concentration of DNA obtained from the original (fresh) saliva samples was slightly higher, this was not significantly different from the concentrations obtained from the pediatric samples (cryopreserved: 4264.1 ng/μL, original (fresh): 4809.3 ng/μL (*p* = 0.5389)). In addition, comparison of the DNA obtained from gingival crevicular fluid also exhibited similar results, which were also not significantly different (cryopreserved: 1762 ng/μL, original (fresh): 2639.5 ng/μL (*p* = 0.2253)).

**Figure 3:**
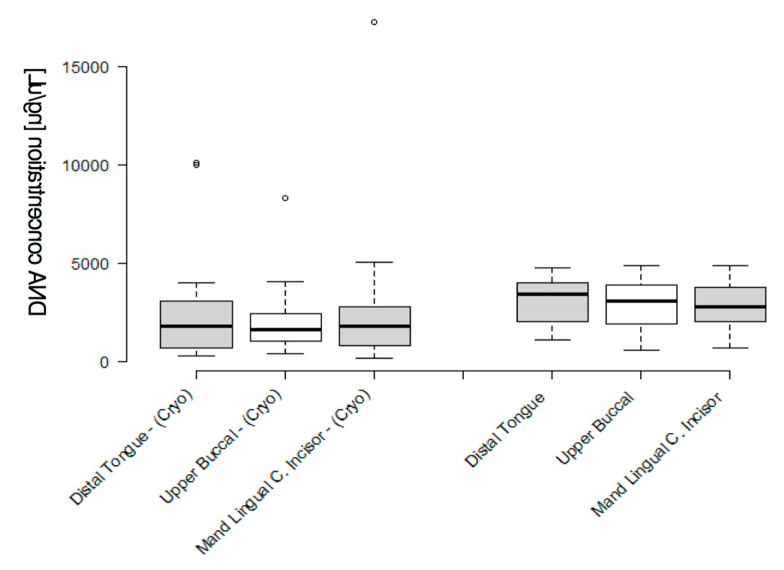
Comparison of the DNA concentrations isolated from the frozen and original (fresh) biofilm samples. Graphic display of representative samples (*n* = 10) demonstrated a slightly broader range of DNA concentrations among the original (fresh) samples compared with cryopreserved samples, with higher averages among the original (fresh) samples. Original (fresh): 2927.3 ng/μL and cryopreserved: 2286.5 ng/μL (*p* = 0.0308).

The concentrations from the surface biofilm samples from the frozen and original (fresh) samples were graphed for comparison (Figure 3). These data demonstrated that the overall DNA concentration isolated from the original (fresh) biofilm samples was higher than, and significantly different from, the concentrations obtained from the cryopreserved samples (*p* = 0.0308). More specifically, the average DNA concentrations from the tongue biofilm, upper buccal molar and lingual incisor from frozen samples (2382 ng/μL, 2048 ng/μL, 2428 ng/μL, respectively) were significantly different from those of the original (fresh) samples (3049 ng/μL, 2838.1 ng/μL, 2894.9 ng/μL, respectively (*p* = 0.0308)).

**Figure 4:**
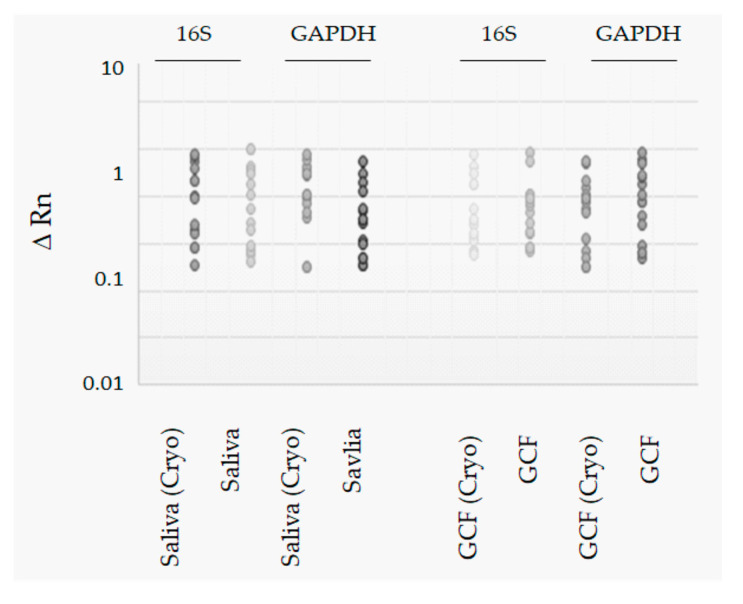
A real-time qPCR screening of cryopreserved and original (fresh) saliva, as well as GCF samples. Screening using universal 16S rRNA bacterial primers and GAPDH human glycolytic pathway primers revealed positive results above the limit of detection (ΔRn = 0.01) with no significant differences in the signal intensity for 16S or GAPDH between the cryopreserved and original samples (*p* = 0.7401).

A real-time qPCR was performed to evaluate the presence of bacterial (16S rRNA) and human (GAPDH) DNA from the salivary and GCF isolates (Figure 4). The qPCR screening revealed that all the samples evaluated harbored bacterial and human DNA of sufficient quantity and quality for a molecular screening greater than the limit of detection (ΔRn = 0.01). No significant differences in the qPCR signal intensity for 16S or GAPDH were found between the cryopreserved and original samples (*p* = 0.7401).

Finally, to determine if there are any study-specific biases within the original sample collection, simple descriptive statistics of the demographic characteristics were assembled for analysis (Table 2). These data demonstrated a significantly lower proportion of females in the study sample versus the overall percentage of females from the pediatric clinic population, from which they were originally obtained (36.2% and 52.8%, respectively (*p* = 0.0012)). In addition, the proportion of samples derived from non-minority (white) patients was significantly lower than the overall percentage from the pediatric clinic population (4.25% and 24.7%, respectively (*p* = 0.00018)).

## 4. Discussion

Although evaluations of DNA isolated from saliva or GCF and site-specific biofilm have been performed in adults [11], the primary goal of this study was to make a quantitative and qualitative comparison to determine the suitability of the DNA from pediatric oral samples for microbial PCR screening. These data provide the first quantitative and qualitative data that suggest sampling of the oral biofilm among pediatric patients using paper points may provide consistent and high quality results from a variety of patients and oral site locations—a novel finding that may expand the range of institutions and research groups that may contribute to the advancement of oral health research [20,21].

In addition, only a very limited number of studies have provided an in depth analysis and comparisons of pediatric and adult biofilm samples [22,23]. This study provides direct comparisons of DNA concentrations not only from unstimulated saliva and GCF, but also from the biofilm derived from several distinct oral sites. Although many efforts have been made to describe changes to the oral biofilm over time in either pediatric or adult patients, these studies may be both cost prohibitive and time consuming—which may delay the discovery and development of relevant findings to help reduce the burden of oral disease [24,25].

As the need for standardized approaches becomes more evident, the validation of standardized protocols and methods using commonly available dental office materials becomes a research priority [26]. This study provides preliminary evidence that contributes to the development of these protocols and methods and also provides comparisons of pediatric and adult samples from different oral biofilm sites. However, this study sample was limited in number due to financial and other site-specific limitations. Future research in this area should include larger comparison samples that may provide more robust evidence for quantitative and qualitative evaluation.

## 5. Conclusions

Many methods are currently available to provide the sampling and screening of saliva and specific sites within the oral cavity, but the validation and comparison of simple and low-cost methods that include paper point sampling and unstimulated saliva collection may suggest that these methods and protocols provide sufficient DNA quality and quantity for molecular screening and other comparison applications. In addition, although heterogeneity will be a constant and consistent feature between patient samples, standardized methods that provide similar and consistent DNA from various oral sites may provide the needed consistency for screening and molecular analysis.

## Figures and Tables

**Table 1:** Quantitative and qualitative analysis of oral site sampling.

Site	Concentration Mean ± SEM	Concentration Median	Statistical Analysis	Purity (A260:A280) Mean ± SEM (Range)	Contamination (A260:A230) Mean ± SEM (Range)
Saliva (*n* = 47)	4264.1 ± 742.2 ng/μL	3038.3 ng/μL	*p* = 0.0348	1.74 ± 0.22 (range: 1.56–2.02)	1.92 ± 0.13 (range: 1.88–2.02)
Dorsum tongue (*n* = 47)	2382.4 ± 445.1 ng/μL	1797.9 ng/μL		1.79 ± 0.19 (range: 1.64–1.99)	1.99 ± 0.17 (range: 1.81–2.13)
Upper buccal (*n* = 47)	2048.4 ± 302.4 ng/μL	1618.3 ng/μL	*p* = 0.5376	1.77 ± 0.26 (range: 1.63–2.11)	1.98 ± 0.11 (range: 1.85–2.06)
Mandibular lingual (*n* = 47)	2428.6 ± 576.5 ng/μL	1795.8 ng/μL		1.82 ± 0.31 (range: 1.55–1.95)	2.01 ± 0.16 (range: 1.90–2.13)
Gingival crevicular fluid (GCF) (*n* = 47)	1771.5 ± 245.9 ng/μL	1669.2 ng/μL	*p* = 0.4805	1.71 ± 0.22 (range: 1.60–1.98)	1.96 ± 0.08 (range: 1.89–2.05)

**Table 2:** Demographic analysis of study samples.

Demographic	Study Sample	Clinic	Statistical Analysis
Sex			
Female	*n* = 17/47 (36.2%)	52.8%	Χ2 = 110.571, d.f. = 1
Male	*n* = 30/47 (63.8%)	47.2%	*p* = 0.0012
**Race/Ethnicity**			
White (non-Minority)	*n* = 2/47 (4.25%)	24.7%	Χ2 = 223.753, d.f. = 1
Minority	*n* = 45/47 (95.75%)	75.3%	*p* = 0.00018
Hispanic	*n* = 35/47 (74.5%)	52.1%	
Black	*n* = 8/47 (17%)	11.8%	
Asian/Other	*n* = 2/47 (4.25%)	11.4%	
**Age**			
Average age	10.26 years	11.34 years	
Age range	5—15 years	0—18 years	
